# A longitudinal examination of the association between nurse staffing levels, the practice environment and nurse-sensitive patient outcomes in hospitals

**DOI:** 10.1186/s12913-015-1198-0

**Published:** 2015-12-04

**Authors:** Janita P. C. Chau, Suzanne H. S. Lo, K. C. Choi, Eric L. S. Chan, Matthew D. McHugh, Danny W. K. Tong, Angela M. L. Kwok, W. Y. Ip, Iris F. K. Lee, Diana T. F. Lee

**Affiliations:** The Nethersole School of Nursing, Faculty of Medicine, The Chinese University of Hong Kong, 8/F, Esther Lee Building, Shatin, NT Hong Kong; Department of Health Sciences, Caritas Bianchi College of Careers, Caritas Institute of Higher Education, 18 Chui Ling Road, Tseung Kwan O, NT Hong Kong; School of Nursing; Center for Health Outcomes and Policy Research, University of Pennsylvania, Room 379, Fagin Hall, 418 Curie Blvd., Philadelphia, Pennsylvania 19104-4217 United States; Infectious Disease Centre, Princess Margaret Hospital, Hospital Authority, Kowloon, Hong Kong; Department of Medicine and Therapeutics, Prince of Wales Hospital, Hospital Authority, NT, Hong Kong

**Keywords:** Health facility environment, Health manpower, Nursing staff, hospital, Nursing service, hospital, Quality indicators

## Abstract

**Background:**

The level of patient safety and outcomes accomplished depends on the quality of care provided. Previous studies found that nurse-to-patient ratio, practice environment, and nursing education were significant predictors of patient outcomes. However, the outcomes measured in previous studies were mainly inpatient mortality and failure-to-rescue rates. Few nurse-sensitive patient outcomes have been measured that quantify nurses’ contribution to patient care. Selecting appropriate outcomes that reflect the clinically relevant effect of nursing care is important. Moreover previous studies were largely cross-sectional and retrospective. These research designs are limited in their ability to explain the casual links between the variables examined. This study is aimed at determining the associations among staffing levels, skill mix of baccalaureate-prepared registered nurses, and practice environment on nurse-sensitive outcomes for medical and surgical patients in public hospitals in Hong Kong.

**Method/designs:**

A multi-method research design will be adopted. The sample includes all medical and surgical wards of four major public hospitals that offer 24-h accident and emergency services. Multiple responses from registered nurses who work in the study wards will be collected over 12 months to examine their individual characteristics and perceptions of the practice environment. A 12-month prospective observational study will be performed to determine the association between nurse staffing levels, the practice environment, and nurse-sensitive patient outcomes including pressure ulcers, falls and restraint prevalence, urinary catheter-associated urinary tract infections, and central line catheter-associated bloodstream infections. Multilevel Cox proportional hazards models will be employed to examine the association between these patient outcomes and the explanatory nursing factors of primary interest (nurse staffing levels, education composition, and practice environment), with adjustment for all patient-, ward- and hospital-level potential confounders (age, sex, diagnosis, comorbidities, level of surgical invasiveness, mortality, length of stay, and type of admission).

**Discussion:**

It is anticipated that knowledge of the association between nurse staffing levels, the practice environment, and nurse-sensitive outcomes will inform the provision of quality and timely patient care. This study will provide a landmark report that is of relevance and importance to patients and to hospital stakeholders and managers, health policy makers, nurses, and educators who advocate patient benefits.

**Trial registration:**

Clinical Trials Registry CCTCTR CUHK_CCT00460. Date of trial registration: 02 July 2015.

## Background

Striving to provide high quality care and optimising patient outcomes are the fundamental goals of healthcare professionals. To attain these goals, it is crucial to maintain a sufficient number of competent nurses in the workforce. However, nursing has been struggling with growing turnover rates and manpower shortages over the past decade. Frontline nurses are frequently reported to have low levels of job satisfaction, high levels of stress or burnout due to heavy workloads, increasing job and public demands, and adverse working conditions [1]. Concerns have been raised over the adequacy of nurses in the provision of quality care and hence its influence on patient outcomes. According to the conceptual relationships delineated by Clarke and Donaldson [2], the quality and quantity of nursing care is influenced by administrative practices, and specifically the number of nurses in the service unit, nurses’ educational qualifications and experience, the care delivery model, and the characteristics of the workplace environment, such as the physical environment, information systems and support services. The level of patient safety and outcomes accomplished depend on the quality of care provided.

Nurse-sensitive measures are defined as ‘the processes and outcomes that are affected, provided, and/or influenced by nursing personnel’ (p.2-112) [2]. In 2004, the National Quality Forum (NQF) endorsed 15 national voluntary consensus standards for nursing-sensitive care. They include patient-centred outcome measures (for example, pressure ulcers, falls and restraint prevalence, urinary catheter-associated urinary tract infections (CAUTI), and central line catheter-associated bloodstream infections (CLCABI); system-centred measures (for example, skill mix, nursing care hours per patient day); the practice environment; and voluntary turnover [3]. Selecting appropriate outcome indicators that are sensitive to nursing interventions is thus imperative to accurately quantify nurses’ contribution to patient care. There is certainly a pressing need for credible research on the effect of nurse staffing conditions and the practice environment on nurse-sensitive patient outcomes. Evidence from such research would be valuable for strategic nursing manpower planning and resource allocation.

### Nurse staffing levels

The level of nurse staffing is commonly measured by the nurse-to-patient ratio, the number of nursing hours per patient day, or the composition or skill mix of nurses of different grades, educational preparation or years of experience [4]. Previous studies have reported mixed results on the influence of nurse staffing levels on patient outcomes [5, 6]. An earlier cross-sectional analysis of data from 10,184 nurses, and 232,342 patients undergoing general, orthopaedic and vascular surgery in 168 hospitals in the United States of America found that an additional patient per nurse was associated with an increase in both the risk-adjusted 30-day mortality and the failure-to-rescue rate of 7 % [7]. Another study analysing data from 5,075,969 and 1,104,659 medical and surgical inpatients, respectively, similarly found that an increase in the proportion and number of hours of care per day by registered nurses (RNs) was associated with shorter hospital stays. The study also reported reduced rates of urinary tract infections (UTI) and upper gastrointestinal bleeding in hospitals with greater number of hours of care by RNs [8]. A systematic review of 43 studies found that richer nurse staffing was related to lower failure-to-rescue rates among surgical patients, and lower inpatient mortality rates and shorter hospital stays among medical patients. However, the evidence on the effect of nursing levels on the incidence of pneumonia and UTI was inconclusive [9]. A systematic review and meta-analysis of 28 studies attempted to stratify the effect of nurse staffing by clinical setting. The results showed that a higher level of staffing with RNs per patient day was associated with decreased rates of unplanned extubation, hospital-acquired pneumonia, respiratory failure, and cardiac arrest in intensive care units patients; lower failure-to-rescue rates in surgical patients; and a shorter duration of hospital stay in both intensive care and surgical patients [10].

### Skill mix of baccalaureate-prepared registered nurses

The entry-level education for RNs in Hong Kong has shifted significantly from hospital-based apprenticeship training to baccalaureate education in the last decade. Four universities are currently offering pre-registration baccalaureate nursing programmes, three of which are funded by the University Grants Committee of Hong Kong [11]. Previous studies document that baccalaureate nursing education is superior to hospital-based training in its provision of comprehensive theory-based education and emphasis on the value of research and evidence-based practice [12]. Graduates from baccalaureate programmes were found to have better critical-thinking and communication skills, which are essential for the expanding roles of nurses [13]. Studies examining the influence of the skill mix of baccalaureate-prepared RNs have recognised its significance for patient outcomes. Two large-scale cross-sectional analyses of patients’ data showed that a higher proportion of RNs prepared with a baccalaureate education was associated with a reduced 30-day inpatient mortality and failure-to-rescue rates [14, 15]. Another study examining 9989 nurses’ data and 228,433 surgical patient records also found that a higher proportion of nurses with a baccalaureate level of education was associated with a shorter duration of hospitalisation among patients with serious mental illness [16].

### Nursing practice environment

The organisational characteristics of the workplace environment have been suggested to mediate the performance of professional nurses and thus affect the quality of care. Organisational characteristics range from the relationships between nurses and managers or physicians, supervisory support and leadership, and nurses’ status or levels of autonomy in practice [17]. It is believed that nurses working within positive environments are encouraged to work to best practice and work effectively with multidisciplinary team members [18], which in turn promotes the quality of care provided and results in better patient outcomes [19]. Increasing numbers of studies support the positive association between nurses’ practice environment and patient outcomes, including 30-day inpatient mortality and failure-to-rescue rates [19, 20]. One study explained that a positive practice environment for nurses is characterised by quality management, an emphasis on the need to develop staff, the presence of frontline managers with supervisory abilities, and good relationships between nurses and physicians [20]. It is interesting to note that in an analysis of secondary data from cancer registry, inpatient claims and administrative and nurse surveys, Friese et al. [19] found that both an unfavourable practice environment and nursing education were significant predictors of failure-to-rescue rates. Similarly, in an analysis of secondary data on 10,184 nurses and 232,342 surgical patients in 168 hospitals, Aiken et al. [20] identified independent inverse relationships among the nurse-to-patient ratio and the proportion of nurses with baccalaureate degrees and adverse patient outcomes. The authors concluded that simultaneously enhancing these two factors through the practice environment would achieve better patient outcomes.

Majority of past studies were conducted in Western countries, and no study has examined the association between nurse staffing levels, the nursing practice environment, and patient outcomes in Hong Kong. To determine the relevance of previous study results to conditions in Hong Kong, the authors conducted a pilot study examining the critical elements of baccalaureate nursing education in preparing graduates to assume a broader scope of nursing practice. A purposive sample of 10 baccalaureate nursing graduates who had assumed advanced practice roles and 12 supervisors of the graduates were interviewed. The findings of the pilot study were consistent with previous findings that a baccalaureate nursing education is important in developing graduates’ critical-thinking, reasoning, writing and organisational skills. The development of these skills in turn helped them to prepare practice guidelines or protocols for their practicing units. The graduates cited practical and emotional support from supervisors and colleagues as a factor that aided their transition to advanced practice roles. After taking up advanced practice roles, the graduates autonomously initiated quality improvement projects to enhance their unit services [21]. However, although the pilot study signifies the relevance of baccalaureate nursing education and an environment that is supportive of patient outcomes, more quantitative evaluations of the effect of nurses on patient outcomes under various nurse staffing conditions and practice environments are warranted.

It is evident from the results of recent studies that nurse staffing levels, the skill mix, and the practice environment are potentially synergistic. It would thus be worthwhile to examine not only their effect as separate entities, but also their interrelationships. The outcomes measured in previous studies were mainly inpatient mortality and failure-to-rescue rates, and few nurse-sensitive patient outcomes have been measured that quantify nurses’ contribution to patient care. Selecting appropriate outcomes that reflect the clinically relevant effect of nursing care is important [22]. Previous studies were also largely cross-sectional and retrospective. These research designs are limited in their ability to explain the casual links between the variables examined. Secondary data, such as past hospital discharge summaries, have been commonly adopted as the chief source of data, which raises concerns about the quality and reliability of the findings [2]. Although it is acknowledged that conducting experimental studies for this type of topic may not be feasible, collecting data prospectively may help enhance the reliability and generalisability of the results. The proposed study will examine the influence of nurse staffing levels, the skill mix of baccalaureate-prepared RNs, and the nursing practice environment on a range of nurse-sensitive patient outcomes among medical and surgical patients in public hospitals in Hong Kong using a prospective design.

## Methods/design

### Aims

This study is aimed to determine the associations among staffing levels, the skill mix of baccalaureate-prepared RNs, and the professional practice environment on nurse-sensitive outcomes for medical and surgical patients in public hospitals in Hong Kong.

### Objectives

1. To examine the association between the staffing levels of RNs and nurse-sensitive patient outcomes for medical and surgical patients;
2. To examine the association between the proportion of RNs educated to baccalaureate level or above upon entry to the profession and nurse-sensitive patient outcomes for medical and surgical patients; and
3. To examine the association between the nursing practice environment and nurse-sensitive patient outcomes for medical and surgical patients.

### Hypotheses

1. Higher RN staffing levels are associated with better nurse-sensitive patient outcomes.
2. A higher proportion of RNs educated to baccalaureate level or higher upon entry to the profession is associated with better nurse-sensitive patient outcomes.
3. A more favourable nursing practice environment is associated with better nurse-sensitive patient outcomes.

### Design and sample

This study will adopt a multi-method research design. The hospital sample will be collected from four major public hospitals that offer 24-h accident and emergency services in Hong Kong and have consented to participate in the study. The clinical wards selected will include adult general medical wards and adult surgical wards (general surgery and orthopaedic wards). There are a total of 34 medical wards and 29 surgical wards in these four hospitals. Multiple responses from RNs who work in the medical wards (around 800 nurses) and surgical wards (around 700 nurses) will be collected over 12 months to examine the individual characteristics of the nurses and their perceptions of the practice environment. A 12-month prospective observational study will be carried out to determine the association between nurse staffing levels and nurse-sensitive patient outcomes.

### Measures and source of data

#### A. Measures of individual nurse characteristics and their perceptions of the practice environment

All RNs, including those in clinical, supervisory, leadership and management roles, who work full time in the study units and have direct patient care responsibilities for 50 % or more of their job will be recruited. Direct patient care responsibilities include activities such as nursing assessment and interventions; health teaching; communication; planning and coordination of care [23].

The following information will be collected every three months over a 12-month period for each study ward:

- Demographic characteristics: Age, sex, work experience as a RN (months), position (RN, advanced practice nurse, nurse specialist, nurse consultant, ward manager, nursing officer) in the study ward, and whether full time or part time.
- Education level and attainment.
- Certification: year of certification, place, type (general/psychiatric/midwife registration).
- Entry-level education to RN: Diploma, pre-registration Bachelor of Nursing degree, pre-registration for Master of Nursing degree.
- Highest credential in nursing: Diploma, pre-registration Bachelor of Nursing degree, pre-registration for Master of Nursing degree, Master degree, Master of Philosophy, Doctor of Nursing, Doctor of Philosophy.

Obtain multiple responses from nurse participants in the study wards over 12 months will address changes in personnel and education attainment over time. The recruitment of experienced or new graduate nurses is often scheduled in summer and every few months. The newly graduated RNs also need to change their rotation three times within two years. Based on our recent study, we anticipate that updating the information from the nurse participants every three months will be able to capture the changes [21].

- Measure of practice environment: The Practice Environment Scale-Nursing Work Index (PES-NWI) [24] will be used to measure the quality of the professional nursing practice environment in each ward. The PES-NWI is a 31-item scale. Respondents rate each item on a scale of 1 (strongly disagree) to 4 (strongly agree) to indicate whether the feature is ‘present in the current job’. The five subscales include nurse participation in hospital affairs; staffing and resource adequacy; nursing foundations for quality of care; nurse manager ability, leadership, and support for nurses; and collegial nurse-physician relations. The PES-NWI is a nationally endorsed nursing care performance standard and has been adopted by the NQF as a nursing-sensitive care performance measure [23]. Cronbach’s alphas for the subscales range from 0.71 to 0.84 based on Lake’s five-factor structure [24]. The Chinese version of the PES-NWI was tested by Chiang and Lin [25] in a study of 842 nurses. The alpha coefficient for the total scale was 0.9, and the alpha for the subscales ranged from 0.65 to 0.87.

#### B. Measures of nurse staffing

The following information on nurse staffing will be collected prospectively from wards, and central nursing division and human resources departments in hospitals.

- RN staffing per unit- shift.
- Number of RNs with a baccalaureate level or higher upon entry to the profession staffing per unit- shift.
- Skill mix information, including the number of full-time and part-time RNs, enrolled nurses and patient care assistants/healthcare assistants working on each shift.
- Nursing care hours per patient day: number of RNs per patient day and number of nursing staff hours (RNs, enrolled nurses, patient care assistants/healthcare assistants) per patient day.

The nurse-to-patient ratio will be computed from the productive direct patient care nursing hours worked by RNs. Productive hours are the number of hours worked by nursing staff with direct patient care responsibilities and exclude paid time off for illness, vacation or continuing education [26].

#### C. Measures of nurse-sensitive patient outcomes

The following nurse-sensitive patient outcomes that are quantifiably influenced by nursing personnel [3] will be retrieved from paper based patient records, nursing documentation, and through the Clinical Data Analysis and Reporting System (CDARS).

- Pressure ulcers prevalence (hospital acquired); falls prevalence (inpatient falls); falls with injury; restraint prevalence; infection rates (hospital acquired): CAUTI; and CLCABI.

The definitions and criteria, data sources and data collection methods for each outcome will be constructed based on the implementation guide for the NQF endorsed nursing-sensitive care performance measures [23].

#### D. Risk-adjustment

Patient-level measures:

We will collect information on patient demographic and clinical information, including age, sex, diagnosis (using the International Statistical Classification of Disease and Related Health Problems 10th Revision), comorbidities, level of surgical invasiveness (mild, intermediate, major), mortality, length of stay and type of admission. This information will be used to measure and control patient acuity across units and hospitals [8]. The information will be retrieved from the admission records, patient notes and discharge summaries of each patient admitted to the study units during the study period. In the 12-month prospective observational study period, we anticipate that there will be around 90,000 episodes of hospital admissions in the medical and surgical wards in the four hospitals [27, 28].

Characteristics of the hospitals, the wards and patient turnover:

We will collect information on the type of hospital, number of beds, teaching status, region and technology (for example, facilities for open-heart surgery and major organ transplants), the types of wards, number of beds, patient bed-days, patient turnover for each shift and patient days (the total number of patient days per ward for a month).

### Data collection procedures

To reduce error in the data abstraction and ensure uniformity of understanding, the two research nurses will undergo training sessions on the abstracting and coding rules. Two of the co-investigators will conduct an audit on a random sample of 5 % of the data collected by the research nurses to verify the data accuracy. Regular meetings will be scheduled to discuss problems with data abstraction and coding. There will be a pilot testing phase before the main study. In this phase, data collection in one medical and one surgical ward will be conducted for two months. Its purposes are to determine the feasibility of the data collection process, the time required and logistics to review records and to retrieve data from the hospital electronic systems. Inter- and intra-observer reliabilities of the questionnaires and data collection forms will be assessed. Necessary adjustments and/or refinements on the data collection process and data collection forms as well as further training for research nurses to facilitate reliable data collection will be made prior to the main study. It is anticipated that a 2-month pilot data collection period would be sufficient to capture enough variety of potential issues encountered in the study.

### Ethical considerations

Ethical approvals have been obtained from the Joint Chinese University of Hong Kong-New Territories East Cluster Clinical Research Ethics Committee and the Kowloon West Cluster Research Ethics Committee. We would uphold the protection of research subjects’ rights and safety through adherence to local laws, Declaration of Helsinki, institutional polices and ICH-GCP. We will also comply with the Hong Kong Personal Data (Privacy) Ordinance. All nurse participants who meet the inclusion criteria will be invited to participate following an explanation of the purpose of the study and an assurance of their rights and freedom to withdraw from the study at any time. If the participants agree to take part in the study, then they will be asked to sign an informed consent form. The questionnaires will be anonymous and will be used for research purposes only. Approval will also be sought to access staffing information, paper based patient records, nursing documentation, and data from the CDARS of each hospital. All identifying information including names and identity card number of the patients will be expunged using correction tape. Each hospital record will be assigned a reference number and the reference number rather than other identifiers will be attached to the data. The data will be analysed anonymously. All information collected will be kept strictly confidential and will be destroyed six years after completion of the study.

### Statistical analyses

The data will be summarised and presented using appropriate descriptive statistics. The normality of the continuous variables will be assessed using skewness and kurtosis statistics and graphically by a Q-Q plot. Appropriate transformations will be made on skewed variables to correct their skewness before being entered into the statistical analyses. As our study design will give three levels of hierarchical data, with levels one, two and three representing individual patients, wards and hospitals, respectively, multilevel modelling will be used to test our hypotheses. Multilevel models can account for intra-correlated hierarchical or clustered data where subjects in the same cluster tend to be more strongly correlated than subjects from different clusters [29]. The nurse-sensitive patient outcomes listed in Table 1 are the outcome measures of the study and will be analysed as dependent variables in separate multilevel models. The unit of analysis for testing our hypotheses will be the patient. A survival analysis approach will be adopted to analyse the time-to-event outcomes. In particular, the status of these outcomes (Yes/No), and the time-to-event for ‘yes’ (case observation) or time-to-hospital discharge, or the time-to-cutoff for the study period (a maximum of 12 months) for ‘no’ (censored observation) since hospital admission will be modelled with this approach. Multilevel Cox proportional hazards models will be employed to examine the association between these patient outcomes and the explanatory nursing factors of primary interest (nurse staffing levels, education composition and practice environment), with adjustment for all patient-, ward- and hospital-level potential confounders (Table 1). These nursing factors will be aggregated to the ward level for analysis. For each included ward, the nursing data will be collected at the start of every three follow-up months (for a total of 12 months) and aggregated to summary values (for example, the proportion of RNs educated to baccalaureate level or above upon entry to the profession). All patients admitted in the same follow-up 3-month period to the same ward will be assumed to have been exposed to the same summary ward-level nursing factors in the Cox models. The association between the nurse-sensitive patient outcomes and the nursing factors will be assessed by the corresponding hazard ratios and their 95 % confidence intervals in the multilevel Cox models. All statistical analyses will be performed using Stata version 12.0 (StataCorp, College Station, Texas, USA). All statistical tests involved will be two-sided, and a *p*-value <0.05 will be considered statistically significant.

**Table 1:** The nurse-sensitive patient outcomes, explanatory nursing factors and potential confounding variables

Variables enter in the multilevel Cox Regression models	Data Level
Nurse-sensitive patient outcomes	
Pressure ulcers (hospital acquired)	Patient
Falls (inpatient falls)	Patient
Falls with injury	Patient
Restraints	Patient
Urinary catheter-associated urinary tract infection	Patient
Central line catheter-associated blood stream infection	Patient
Explanatory nursing factors	
Education composition of registered nurses: the proportion of registered nurses educated to baccalaureate level or higher upon entry to the profession	Ward
Registered nurses staffing level: full-time equivalent employment	Ward
Practice environment:	Ward
Nurse participation in hospital affairs (mean subscale score)	
Staffing and resource adequacy (mean subscale score)	
Nursing foundations for quality of care (mean subscale score)	
Nurse manager ability, leadership, and support of nurses (mean subscale score)	
Collegial nurse-physician relations (mean subscale score)	
Potential confounding variables	
Patients’ characteristics: age, sex, diagnosis, comorbidities, level of surgical invasiveness, mortality, length of stay and type of admission.	Patient
Characteristics of the wards and patient turnover: types of wards, number of beds, patient bed-days and patient turnover for each shift and patient days	Ward
Characteristics of the hospitals: type of hospital, number of beds, teaching status, region, and technology.	Hospital

### Trial status

The research ethics approvals and the institutional approvals were granted. The recruitment of research nurses for conducting the study is in progress. Data collection is expected to begin shortly.

## Discussion

Ensuring vigilant human resource planning and continuously improving the quality of the practice environment for nurses are crucial steps to securing the stable and well-prepared nursing workforce that is essential to sustaining the provision of high-quality healthcare services for Hong Kong people. The proposed study will be the first to examine the influence of nurse staffing levels, the skill mix of baccalaureate-prepared RNs and the professional practice environment on medical and surgical patients’ outcomes in Hong Kong. The study will lay the groundwork for determining the operational definitions and methods for measuring the appropriate quantity of nursing time and the optimal type of practice environment for various healthcare situations in Hong Kong, and will thus inform future study designs. It is anticipated that the enhancement of the three factors that will be examined could promote a more caring environment in which patients receive quality and timely care from a sufficient number of well-qualified nurses. The study will be a landmark report of relevance and importance to patients and to hospital stakeholders and managers, health policy makers, nurses and educators who advocate patient benefits. The study will also guide the development of better data systems for determining the influence of nurses on patient outcomes.

According to its annual plans 2013/14 and 2014/15, one of the Hospital Authority’s service priorities is to allay staff shortage and high turnover, and ensure service quality and safety [1, 30]. Rather than recommending a minimum nurse staffing level, the Hospital Authority conducted an annual patient dependency assessment to evaluate the sufficiency with which service demands are met. In addition to the number of admissions and patient bed-days, the nursing time required is also involved in assessments of patients’ nursing care demands [31]. The results of the proposed study will help to identify the current nurse staffing patterns and supplement valuable data for health policy makers and hospital stakeholders to determine the number of baccalaureate-prepared RNs needed to meet the estimated future demand across healthcare services. Only when these figures are available can the systematic planning of RN recruitment and resource allocation to match the needs of professional development be developed.

Although the study will focus on medical and surgical patients, managers of other specialties could use it as a reference to estimate the RN-to-patient ratio and skill mix of baccalaureate-prepared RNs according to their needs at the unit level. Such assessments would in turn help inform decisions about the deployment of RNs with nursing degrees upon entry to the profession and nurses with other educational qualifications on shifts or units. Given the heightened pressure on healthcare budgets, this study will provide justification for investment in RNs and directions for new strategies that ensure effective collaboration between nurses with different educational backgrounds. In line with the systematic workforce planning and development that hospitals must undertake, the higher education sector could use the study results to plan the target number of baccalaureate nursing degree places to be offered. Such planning is crucial not only to ensure an adequate supply of well-prepared nurses to meet service needs, but also to safeguard the timely strategic development of higher institutions, including the quality and academic standards of nursing programmes.

The study will help to identify the essential attributes of a positive and productive practice environment that keeps patients well cared for and boosts staff morale. Such evidence will indicate directions for organisational change, and specifically the re-structuring and re-engineering of nurses’ working environments. It is expected that nurses practicing in productive environments will be more autonomous in the provision of high-quality nursing care, which will result in positive patient outcomes. Improving the practice environment, in tandem with the manipulation of nurse staffing levels and the skill mix at the organisational level, will help ensure high-quality care while avoiding nurses becoming overloaded. The study will be a unique opportunity to indirectly enhance nurses’ job satisfaction and avoid burnout, and ultimately improve nurse retention in the profession [17]. Another long-term impact will be the amassing of evidence on the extent to which RNs contribute to improving patient outcomes. Such evidence is crucial in acknowledging nurses’ roles in this regard, and will lead to greater nurse professionalism; a critical element in strengthening the nursing workforce [32].

## Abbreviations

CAUTI: Catheter-associated urinary tract infections
CDARS: Clinical data analysis and reporting system
CLCABI: Central line catheter-associated bloodstream infections
NQF: National Quality Forum
PES-NWI: Practice Environment Scale-Nursing Work Index
RNs: Registered nurses
UTI: Urinary tract infections
